# *IMPA2* polymorphisms and risk of ischemic stroke in a northwest Han Chinese population

**DOI:** 10.18632/oncotarget.12133

**Published:** 2016-09-20

**Authors:** Qiaoya Ma, Ying Yang, Yuyan Na, Tianbo Jin, Yidong Xue, Yuting Shi, Chen Li, Wanggang Zhang

**Affiliations:** ^1^ Department of Geriatrics Neurology, The Second Affiliated Hospital of Xi'an Jiaotong University, Xi'an, Shanxi, P. R. China; ^2^ Department of Orthopaedics, Graduate School of Inner Mongolia Medical University, Hohhot, Inner Mongolia Autonomous Region, P. R. China; ^3^ Department of Biochemistry, School of Life Sciences, Northwest University, Xi'an, Shanxi, P. R. China; ^4^ Department of Neurology, The Affiliated Hospital of Yan'an University, Yan'an, Shanxi, P. R. China; ^5^ Department of Medical Oncology, Graduate School of Inner Mongolia Medical University, Hohhot, Inner Mongolia Autonomous Region, P. R. China; ^6^ Department of Hematology, The Second Affiliated Hospital of Xi'an Jiaotong University, Xi'an, Shanxi, P. R. China

**Keywords:** ischemic stroke (IS), single nucleotide polymorphisms (SNPs), inositol monophosphatase 2 (IMPA2), case-control study

## Abstract

Genetic association analysis has suggested that *IMPA2* is a susceptibility gene for ischemic stroke (IS). To explore the association between *IMPA2* polymorphisms and the risk of IS in a Han Chinese population, candidate gene association was performed using data from a case-control study of 488 IS patients and 503 control subjects. Odds ratios (ORs) and 95% confidence intervals (CIs) were used to assess the association, and associations were evaluated under dominant, recessive, and additive genetic models using PLINK software. There was a statistically significant difference in the “TC” genotype frequency of the *IMPA2* polymorphism rs589247, between cases and controls (50.0% vs. 45.3%). Under the dominant model, rs589247 was associated with an increased risk of IS (OR=1.32, 95%CI: 1.01-1.73; *P*=0.040). There were no other associations between any of the seven additional *IMPA2* polymorphisms and IS risk. This study is the first to find a correlation between an *IMPA2* polymorphism and IS risk in a northwest Han Chinese population. These results may help to elucidate the molecular pathogenesis of this disease, and could potentially be used to predict IS risk. However, further studies are still needed to validate this association in other populations and with larger sample sizes.

## INTRODUCTION

Ischemic stroke (IS) is a leading cause of death and disability [1]. Worldwide, approximately 15 million individuals suffer from stroke every year, and at least 80% of them are IS patients. IS is the result of interrupted blood flow within the area of an occluded blood vessel, causing local brain tissues to become short of oxygen, ending in malacia and necrosis [2]. There are various pathophysiological causes of IS, including atherothrombosis and embolism [3]. While IS can affect both men and women at any time of life, it is more prevalent in men over 45 years of age [4]. In addition, some rare IS syndromes, such as cerebral autosomal dominant with subcortical infarcts and leukoencephalopathy (CADASIL) syndrome, Mitochondrial encephalomyopathy, lactic acidosis, and stroke-like episodes (MELAS) syndrome, and Fabry disease, mainly occur in young and middle-aged men [5]. Because of the high cost of IS treatment and medical rehabilitation, the preferred approach would be primary prevention through the identification of genetic and environmental contributions to IS risk.

IS is a complex and heterogeneous disease influenced by both genetic and environmental factors [6, 7]. Common risk factors for IS are older age, hypertension, diabetes mellitus, hyperlipidemia, smoking, and atrial fibrillation [8]. Beyond these risk factors, many candidate genes for this illness have been investigated with numerous significant associations, such as variants in *APOD* in Swedish populations [9], *KCNK17* in Spanish populations [10], and *APOA5* in Han Chinese populations [11, 12]. These candidate genes may have a more prominent influence on disease onset when environmental factors have not yet had sufficient time to modify the phenotype. In complex diseases, population-based association studies provide more statistical power than family-based linkage studies [13]. Therefore, in the present study, candidate gene association was adopted to find genetic polymorphisms implicated in IS.

*IMPA2*, located on chromosome 18p11.2, encodes *myo*-inositol monophosphatase 2, which is involved in the phosphatidylinositol signaling pathway [14]. This gene was identified as a febrile seizure susceptibility gene [15], but is also associated with neuropsychiatric disorders, such as bipolar disorder. Carriers of the “AA” genotype for *IMPA2* rs669838 are also more likely to have a history of suicide attempts in comparison to C-allele carriers [16], and *IMPA2* or a nearby gene may contribute to the overall genetic risk for schizophrenia [17]. In 2007, a genome-wide association study (GWAS) showed that *IMPA2* polymorphisms are associated with IS in an American population [18]. We wanted to know whether these findings could be reproduced in a Han Chinese population. Therefore, eight *IMPA2* SNPs (rs7506045, rs589247, rs669838, rs636173, rs3786385, rs1020294, rs1250171, and rs613993) that had been investigated for bipolar disorder and/or IS with minor allele frequencies (MAF) >5% in a Han Chinese Beijing population were selected to investigate their potential correlations.

## RESULTS

We used candidate gene association analysis to determine the genetic susceptibility of single nucleotide polymorphisms (SNPs) in the *IMPA2* gene with IS risk in a Han Chinese population. Eight SNPs in the *IMPA2* gene were successfully genotyped in IS patients and neurologically normal controls. Table 1 shows the allele distributions of the eight SNPs and their associations with IS risk. All eight SNPs conformed to Hardy-Weinberg equilibrium in controls with a value of *P* >0.05. There was a statistically significant difference in “TC” genotype frequency of rs589247 between cases and controls (50.0% vs. 45.3%). As shown in Table 2, the “TC” genotype of rs589247 was associated with an increased risk of IS (OR= 1.33, 95%CI: 1.00-1.77, *P*= 0.048).

**Table 1:** Allele distribution in cases and controls and odds ratio estimates for ischemic stroke

SNPs	Chromosome	Position	Minor allele	Minor allele distribution		OR(95%CI)	*P*	HWE *P* value
				Cases	Controls			
rs7506045	18p11.21	11987272	T	240 (0.25)	266 (0.26)	0.91 (0.74-1.11)	0.358	0.566
rs589247	18p11.21	11992725	T	448 (0.46)	424 (0.42)	1.16 (0.98-1.39)	0.092	0.120
rs669838	18p11.21	11994598	C	444 (0.46)	422 (0.42)	1.15 (0.97-1.38)	0.112	0.120
rs636173	18p11.21	11996337	G	444 (0.46)	418 (0.42)	1.18 (0.99-1.41)	0.070	0.199
rs3786285	18p11.21	12008847	G	424 (0.44)	448 (0.45)	0.96 (0.81-1.15)	0.199	0.126
rs1020294	18p11.21	12017343	G	186 (0.19)	167 (0.16)	1.18 (0.93-1.48)	0.165	0.197
rs1250171	18p11.21	12027028	C	214 (0.22)	231 (0.23)	0.94 (0.76-1.16)	0.564	0.313
rs613993	18p11.21	12028580	A	206 (0.21)	227 (0.23)	0.92 (0.74-1.13)	0.419	0.307

HWE: Hardy-Weinberg equilibrium; OR: odds ratio; 95%CI: 95% confidence interval.

*P* values were calculated from Chi-square test/Fisher's exact test.

**Table 2:** Association between rs589247 and ischemic stroke risk under multiple genetic models

SNP	Models	Genotype	Genotype frequency		OR (95%CI)	*P^a^*	*P^b^*
			Control	Case			
rs589247	Genotype	CC	177	142	1.000		
		TC	228	244	1.33 (1.00-1.77)	0.048*	1
		TT	98	102	1.30 (0.91-1.85)	0.150	1
	Dominant	TT+TC	326	346	1.32 (1.01-1.73)	0.040*	1
		CC	177	142	1.000		
	Recessive	TT	98	102	1.09 (0.80-1.49)	0.578	1
		TC+CC	405	386	1.000		
	Additive	-	-	-	1.16 (0.97-1.38)	0.098	1

SNPs, single-nucleotide polymorphisms; OR, odds ratio; 95%CI, 95% confidence interval.

a *P* values were calculated from unconditional logistic regression analysis.

b *P* values were adjusted by Bonferroni correction.

* *P*≤0.05 indicates statistical significance.

Under several genetic models, we measured the potential association between the polymorphisms and IS susceptibility. As shown in Table 2, rs589247 was associated with an increased risk of IS under a dominant model (OR=1.32, 95%CI: 1.01-1.73; *P*=0.040). Apart from this variant, there was no evidence of significant associations between the other 7 SNPs and IS risk.

In addition to SNP-based association analysis, we also performed haplotype analysis for the *IMPA2* gene and found strong linkage of these eight candidate SNPs. Supplementary Figure 1 shows linkage disequilibrium measures and the haplotype block maps for the candidate SNPs across the *IMPA2* gene. In Block 1, there was a 9 kb genomic region involving four SNPs (rs7506045, rs589247, rs669838, and rs636173) and in Block 2, there was a 1 kb genomic region including two SNPs (rs1250171 and rs613993). Haplotype analysis of the *IMPA2* gene did not reveal any statistically significant associations between any of the five haplotypes and IS risk (Table 3).

**Table 3:** *IMPA2* haplotype frequency and the association with ischemic stroke risk

Haplotype block	Haplotype frequency		OR (95%CI)	Chi-square	*P*
	cases	Controls			
rs7506045-rs589247-rs669838-rs636173					
C-T-C-G	0.454	0.412	1.18 (0.99-1.40)	3.402	0.065
T-C-A-A	0.243	0.259	0.92 (0.75-1.13)	0.688	0.407
C-C-A-A	0.293	0.316	0.90 (0.74-1.09)	1.162	0.281
rs1250171-rs613993					
C-A	0.210	0.227	0.91 (0.73-1.12)	0.789	0.374
T-G	0.780	0.771	1.06 (0.85-1.30)	0.243	0.622

OR, odds ratio; 95%CI, 95% confidence interval.

*P* values were calculated from unconditional logistic regression analysis.

## DISCUSSION

To investigate whether SNPs in *IMPA2* influence IS susceptibility in a Han Chinese population, we performed a candidate gene association study using a case-control design. The association of *IMPA2* with IS was previously reported in a GWAS by Matarin et al. [18] We found a significant association with IS risk was for rs589247 under both a genotype model and a dominant model. This result suggests that rs589247 in the *IMPA2* gene may confer risk for IS under a dominant model.

*Myo*-inositol monophosphatase 2, which is encoded by the *IMPA2* gene, converts inositol phosphate into inositol through dephosphorylation. Inositol is an essential intracellular signaling molecule in second messenger pathways that may exert an insulin-like effect and improve blood pressure, cholesterol, high-density lipoprotein, and triglyceride levels which are all involved in the pathogenesis of IS [19]. We found that the *IMPA2* rs589247 polymorphism was associated with an increased risk of IS. Thus, it is possible that this SNP inhibits *Myo*-inositol monophosphatase expression, thereby contributing to a predisposition for IS.

In the GWAS of IS patients conducted by Matarin et al., there was no significant association between rs7506045 and IS susceptibility in an American population after Bonferroni correction [18]. Three years later, Domingues-Montanari and colleagues analyzed the same relationship in a Spanish Caucasian population comprised of IS patients and healthy controls, and found that *IMPA2* rs7506045 was associated with a statistically significant increase (1.57-fold) in IS susceptibility under an additive model [10]. However, we did not observe any significant relationship between *IMPA2* rs7506045 and IS risk using an additive model analysis (OR=0.91, 95% CI: 0.75-1.11; *P*=0.363). This difference in results may be due to the different ethnicity of our subjects, and/or to inadequate statistical power. Ding et al. previously carried out a study to confirm the GWAS variants reported by Matarin et al. in a Han Chinese population from Wuhan, China [20]. Consistent with our findings, they did not find a significant association of rs7506045 with IS risk under an additive model (OR=1.21, 95% CI: 0.95-1.53; *P*=0.128). Taken together, these data provide support for the notion that rs7506045 in *IMPA2* might have different effects on IS risk in different populations.

To our knowledge, this study is the first to investigate the potential association of *IMPA2* polymorphisms with IS risk in a northwest Han Chinese population, and is the first to explore several novel variants in *IMPA2* in IS. We found rs589247 was associated with increased risk of IS under a genotype model and a dominant model. However, none of our results remained significant after Bonferroni correction. This correction might be overly conservative, or the limited sample size in our study might cause a negative result. In addition to the small sample size, our study has other limitations that should be mentioned. First, we did not take into consideration the association of conventional risk factors, such as smoking, hypertension, obesity, elevated triglyceride and cholesterol levels, diabetes mellitus and dyslipidemia, for IS risk with *IMPA2* polymorphisms. Second, there may be other SNPs on 18p11.21 that are related to IS risk but were not assessed for their potential associations.

In conclusion, our study provides evidence for a potential association of rs589247 in the *IMPA2* gene with increased risk of IS. However, it is unclear whether this finding will be reproduced in other populations. Therefore, more detailed functional and epidemiological studies in larger populations are still needed to validate the association of *IMPA2* variants in the development of IS.

## MATERIALS AND METHODS

### Study subjects

A total of 488 patients (mean age: 63.96 years, 66.6% male) with IS were enrolled from the Geriatric Neurology Department of the Second Affiliated Hospital of Xi'an Jiaotong University and the Neurology Department of the Affiliated Hospital of Yan'an University from February 2013 to December 2015. All individuals involved in this study gave written consent for genetic analysis. All participants underwent a detailed medical history interview and physical examination. Inclusion and exclusion criteria were as follows: (1) Brain computed tomography (CT) and magnetic resonance imaging (MRI) combined with history interview and neurological examination were used to confirm the diagnosis of IS according to the International Classification of Disease; (2) Blood samples were drawn from ethnic Han Chinese population who were genetically unrelated within Northwest China; (3) None had a history of other types of stroke (transient ischemic attack and subarachnoid hemorrhage), cerebral vein thrombosis, Parkinson's disease, Alzheimer's disease, bipolar disorder, or brain aneurysm; (4) Subjects with cardiogenic thrombosis and atrial fibrillation which may contribute to the development of IS were also excluded. As a control group, 503 ethnically and geographically matched and neurologically normal inpatient individuals were randomly recruited from various departments of the Second Affiliated Hospital of Xi'an Jiaotong University. The study protocol was approved by the ethical committee of the Second Affiliated Hospital of Xi'an Jiaotong University and the Affiliated Hospital of Yan'an University.

### DNA isolation and genotyping

Peripheral blood (5 ml) was collected from each subject into tubes containing ethylenediaminetetraacetic acid to prevent clotting. After centrifugation, the samples were kept at −80°C until further use. Genomic DNA was extracted from peripheral blood samples using the GoldMag-Mini Purification Kit (GoldMag Co. Ltd. XI'an city, China) according to the manufacturer's instructions. DNA concentrations were measured using a NanoDrop 2000 (Thermo Scientific, Waltham, Massachusetts, USA). DNA was diluted using QIAgility to a final concentration of 20 ng/μl. Eight SNPs in the *IMPA2* gene with a minor allele frequency (MAF) >5% in a Han Chinese Beijing population were selected for genotyping using the HapMap database. Sequenom Mass ARRAY Assay Design 3.0 Software (Sequenom Co. Ltd, San Diego, California, USA) was used to design a Multiplexed SNP MassEXTEND assay. PCR primers for the eight selected SNPs are listed in Supplementary Table 1. SNP genotyping was carried out by using the Sequenom Mass ARRAY RS1000 [21]. Data were managed and analyzed with SequenomTyper 4.0 Software (Sequenom Co. Ltd) [21, 22].

### Statistical analysis

Statistical analysis was conducted using Microsoft Excel and SPSS 16.0 (SPSS, Chicago IL USA). Statistical significance was set at a two-sided *P*< 0.05. Fisher's exact tests were used to test for deviation from Hardy-Weinberg equilibrium. The distributions of genotype and allele of each SNP were compared using unconditional logistic regression analysis and Chi-square/Fisher's exact tests in cases and controls. Odds ratio (OR) and 95% confidence intervals (CI) were used to assess the effect of each polymorphism and IS risk [23]. All association analysis were conducted under three genetic models: dominant, recessive, and additive using PLINK software (http://pngu.mgh.harvard.edu/purcell/plink/). In addition, the SHEsis software platform (http://analysis.bio-x.cn/myAnalysis.php) and Haploview software package (version 4.2) were used to analyze and visualize patterns of linkage disequilibrium (LD) and haplotype maps [24, 25]. Haplotype blocks were identified using the solid spine of LD method in Haploview.

## SUPPLEMENTARY FIGURE AND TABLE


