# Supplementary material for: IMPA2 polymorphisms and risk of ischemic stroke in a northwest Han Chinese population
Source: Oncotarget. 2016 Sep 20;7(46):75273–8. doi: 10.18632/oncotarget.12133 (PMC5342739; doi:10.18632/oncotarget.12133)
Supplement: Supplementary file 1 [file oncotarget-07-75273-s001.pdf]

IMPA2 polymorphisms and risk of ischemic stroke in a northwest Han Chinese population

SUPPLEMENTARY FIGURE AND TABLE

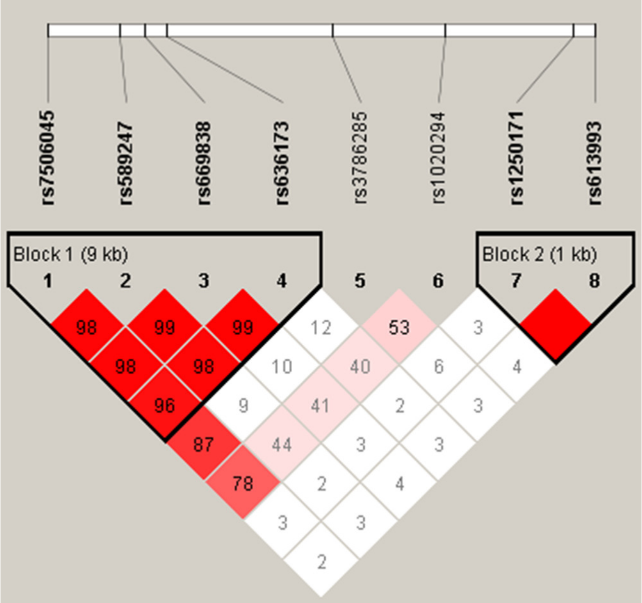

Supplementary Figure S1: Haplotype block map for SNPs in the *IMPA2* gene.

Supplementary Table S1: Primers used for the analysis of *IMPA2* polymorphisms

SNPs	First PCRP (5′→3′)	Second PCRP (5′→3′)	UEP SEQ (5′→3′)
rs7506045	ACGTTGGATGTT CCCAGCTGACATAGATGC	ACGTTGGATGGAATGAATAG TGATGTACTC	GGCACAATGGGAACTTC
rs589247	ACGTTGGATGGCTTA GTTAGCGAGTAGCTG	ACGTTGGATGTCCCTCAGTCT TAGAGATTT	aAGGATACCTAACTCATTGC
rs669838	ACGTTGGATGGCAC ACTGAGCTCACAGTTA	ACGTTGGATGCTGAGTGTATAA ATACCAG	ttTCCTTTTGTGTCACCTTATC
rs636173	ACGTTGGATGTTG GGATGTGTTCTGTGAGC	ACGTTGGATGACATCACATCAC TGTACTGC	ccgGTGAGCAGCGGGAAGCC
rs3786285	ACGTTGGATGTGA GTCCCTCTGCAAACAAG	ACGTTGGATGAGAACATTGTCT CGGTCAGG	ggaacCAAGGCCCCCTACAATC CTCAC
rs1020294	ACGTTGGATGAGAGA GCAGTCTGTTTTTGG	ACGTTGGATGAATCCCAGGCATC AGGTCAC	GTTTTTGGAGACACGC
rs1250171	ACGTTGGATGAAAAG GGACACTTGGACCTG	ACGTTGGATGTTACTGCACTCAA TGGACCC	GAGATTTCCTGGGTGAG
rs613993	ACGTTGGATGAATGC CTTGTGCCATGACAG	ACGTTGGATGTGTCACGTTCAGTA GAGGAG	ATGCAGCAACCAGAGAA

SNPs, single-nucleotide ploymorphisms; PCRP, PCR primer; UEP, Un-extended mini-sequencing primer.
